# Manipulation of a clubfoot prior to a Ponseti method decreases the need for tenotomy!

**DOI:** 10.1097/MD.0000000000029910

**Published:** 2022-08-12

**Authors:** Fanjalalaina Malinirina Ralahy, Josué Lili Andriamasinilaina, Katherine Stannage, Jessie Lucey Gray, Duval Gaëtan Solofomalala

**Affiliations:** a University Hospital (CHU) in Fianarantsoa; b Medecine Faculty of Antananarivo.

**Keywords:** clubfoot, manipulation, Pirani score, Ponseti, tenotomy

## Abstract

The combination of the Ponseti method with functional treatment produces better results and may reduce the need for surgery. The objective of this study was to assess the impact of manipulation of a congenital equinovarus clubfoot performed before correction by the Ponseti method.

This was a cohort study of children <5 years treated with the Ponseti method followed over a minimum period of 1 year. Each foot was treated according to the Ponseti method. The study parameters were as follows: age, gender, concept of previous treatment, previous treatment with manipulation, the degree of deformation according to the Pirani score, the laterality of the deformation, the number of casts required and the time needed for correction, the evolution of the Pirani score before each correction by plaster, the use or not of tenotomy and the Pirani score at the end of the correction session, the evolution of the Pirani score when wearing an abduction boot.

The series comprised a total of 68 feet. The average age of the children was 15.5 months. Regarding the deformity, 29 children presented a severe or very severe deformity. Before the treatment, 16 children received regular handling massage. Feet that had received manipulation prior to correction were the least exposed to tenotomy (*P* = .009). For the children who did not require a tenotomy, all the feet had a Pirani score of zero after the fourth week of wearing the splint. We noted a rapidly decreasing in the Pirani score of the feet, which did not require an tenotomy compared with other feet (Kolmogorov-Smirnov test: D = 0.61; *P* = .01).

The combination of functional treatment with the Ponseti method reduces the need for tenotomy.

## 1. Introduction

The deformity of a congenital equinovarus clubfoot is caused primarily by musculoligamentous retraction and dyskinesia and secondarily by osteoarticular disorganization.^[1]^ The current therapeutic possibilities act on these anomalies at different levels.^[2]^ The Ponseti method achieves ligament elongation by sequentially correcting the joint arrangement. Functional treatment works on muscle kinesis and ligament retraction. Surgery, in addition to ligament release and tendinomuscle transfers, corrects the osteoarticular deformity. Surgery is accompanied by conservative treatments, which remain the benchmark, to correct residual deformities.^[3]^ However, the combination of the Ponseti method with functional treatment produces better results and may reduce the need for surgery.^[4]^ This therapeutic combination puts physiotherapy in addition to Ponseti, but no study reports the results of Ponseti after physiotherapy. The objective of this study was to assess the impact of manipulation of a congenital equinovarus clubfoot performed before correction by the Ponseti method.

## 2. Patients and Method

The study was carried out at the clubfoot treatment center using the Ponseti method of the University Hospital Center (CHU) in Fianarantsoa (Madagascar). This was a cohort study of children treated with the Ponseti method followed over a minimum period of 1 year and <5 years. The study period was 12 months and 17 days, running from April 13, 2018 (date of first consultation of the first child) to May 30, 2019 (date of assessment of the last child). Children included in the study are those under 5 years of age with idiopathic congenital equinovarus clubfoot, treated according to the Ponseti method. The sampling was carried out thoroughly after the parents’ censorship. Children who underwent foot surgery before treatment and those who did not complete care in the center were excluded from the study.

The child’s initial examination assessed the morphology and degree of deformity of the foot and identified associated abnormalities. The study included children with congenital equinovarus clubfoot who completed all steps of the Ponseti method and who received abduction boots. Parental consent was sought before including children in the study. Children considered to have benefited from massage therapy prior to Ponseti correction are those who received manual deformity reduction sessions associated with putrefaction along the length of the muscle and triceps tendon. The session should last at least 15 minutes with a frequency of 2 to 3 times a day for a period of at least 3 months. This manual reduction was carried out by a qualified third party or not.

Each foot was treated according to the Ponseti method. The casts were changed every week. The correction was carried out gradually with the aim of first obtaining a correction of supination, adduction, and varus in the fifth plaster cast (Pirani score for the forefoot = 0). If this goal was reached before the sixth plaster cast, we proceeded directly to the correction of the equinus. Conversely, if the correction of supination or adduction or varus was not achieved, the correction was continued beyond the fifth plaster cast.

Correction using the Ponseti technique was initiated during the first consultation at the center. The correction was carried out by a team made up of an orthopedic surgeon, a physiotherapist, and 2 specialized nurses, who receive regular training and annual updating by Australian colleagues.

The correction session ended with correction of the equinus. Achilles tendon tenotomy was performed if the Pirani score of the hindfoot was ≥1.5/3. We have other hand, the tenotomy was not performed if the Pirani score of the hindfoot was ≤1. After the tenotomy, if wearing an abduction splint was not comfortable especially for children of walking age, a new correction session was made.

The study parameters were as follows: age (months), gender (female, male), concept of previous treatment, previous treatment with manipulation, the degree of deformation according to the Pirani score (minimal, moderate, severe, very severe), the laterality of the deformation (bilateral or unilateral), the number of casts required and the time needed for correction, the evolution of the Pirani score before each correction by plaster, the use or not of tenotomy and the Pirani score at the end of the correction session, the evolution of the Pirani score when wearing an abduction boot. The study protocol was validated by the ethics committee of Hospital in Fianarantsoa. The data were recorded and processed using Epi-info 7 software.

## 3. Results

**Figure 1.**
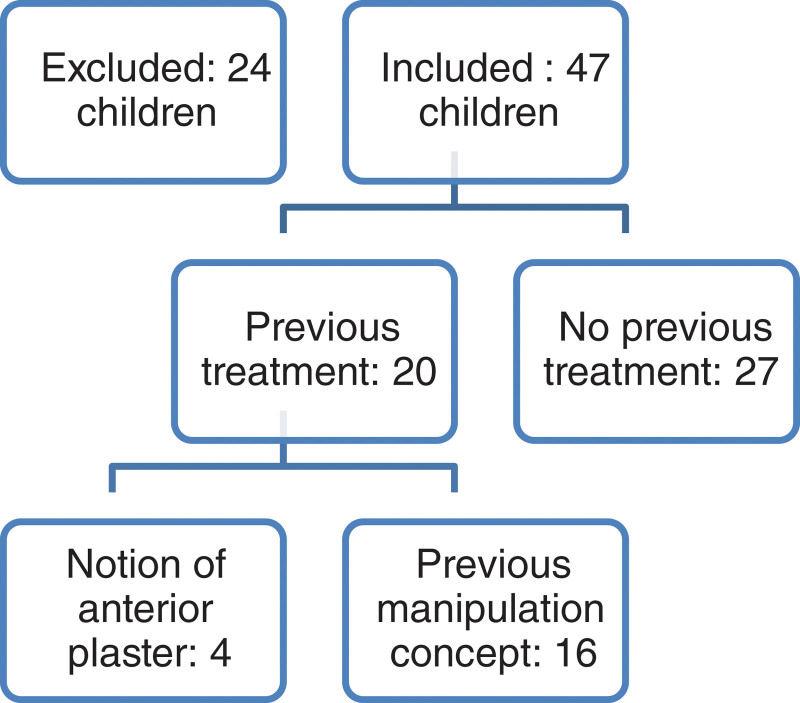
Childrens flowchart.

Over a recruitment period of 4 months, 71 children were enrolled for the study, on which 9 were excluded because of a previous surgery and 15 were over 5 years old. Of the 47 children, 21 presented with bilateral clubfoot. The series thus comprised a total of 68 feet (Figure 1).

The sex ratio was 3.5 (male/female). The average age of the children was 15.5 months (5 days–5 years) with a median age of 11 months. The most represented age group was between 2 and 4 years for 21 children (44.6%), between 6 and 11 months was of 10 children (21%), between 12 and 23 months was 10 children (21%), and between 5 days and 5 months was of 6 children (12.7%). A family history of clubfoot was found in 8 children (17%).

Regarding the deformity, 29 children presented a severe or very severe deformity and only 2 children had minimal deformity (Table 1). Among the 47 children, 27 children (43 feet) had no history of previous treatment, 20 had received previous treatments including, 16 children (21 feet) by regular handling massage and 4 children (4 feet) had been treated with casting sessions.

**Table 1:** Distribution of children according to age and severity of deformity.

	Degree of deformation				
	Minimal	Moderate	Severe	Very severe	Total
5 d–5 mo	1	2	1	2	**6**
6–11 mo	1	3	2	4	**10**
12–23 mo	–	3	1	6	**10**
2–5 yrs	–	8	4	9	**21**
**Total**	2	16	8	21	**47**

**Figure 2.**
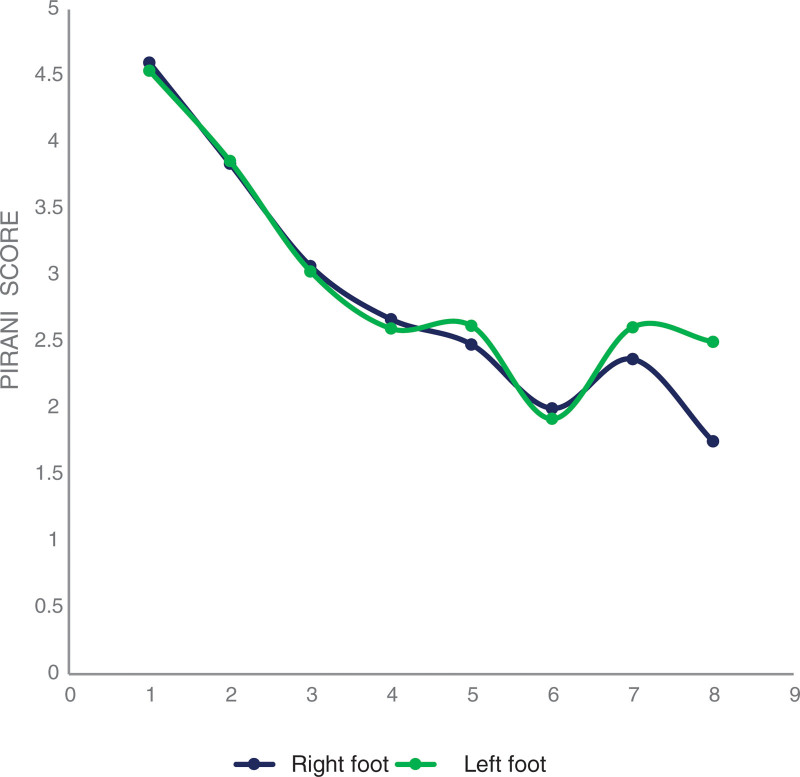
Evolution curves of the weekly Pirani score during the correction.

The number of casts needed for the correction phase ranged from 2 to 8 in the 2 groups of patients (with a history of previous treatment and no history of previous treatment). Of the 47 children, 4 required a second correction session, they were children over 3 years old and had no history of manipulation massage who presented residual equinism after tenotomy. The mean number of casts required for correction in patients with and without previous treatment was 5 ± 1.5 (39.2 ± 10.5 days) and 5 ± 1.3 (40.6 ± 7 days), respectively. The mean number of casts in children who received massage treatment before admission was 6 ± 1.4. For those who received the plaster treatment, the average plaster number was 5 ± 1.5. The general evolution of the mean Pirani score was favorable, ranging from 4.2/6 in the first plaster to 1.6/6 in the sixth plaster (Figure 2). Of the 68 feet, 48 had required percutaneous tenotomy while 19 did not. For the children who did not require a tenotomy, all the feet had a Pirani score of zero after the fourth week of wearing the splint.

Feet that had received manipulation prior to correction were the least exposed to tenotomy (10 nontenotomized feet/11 tenotomized feet) (*P* = .009) (Table 2). In contrast, the 9 children with a history of correction by plaster (4 feet) all required a tenotomy. For children who did not require Achilles tenotomy, the mean Pirani score of the feet was 0/6 after week 3 of splinting. For feet requiring Achilles tenotomy, the mean Pirani score was 0.7/6 at week 7.

**Table 2:** Relationship between “manipulation” and “Achilles tenotomy.”

		**No tenotomy**	**With tenotomy**	**Odds ratio**	**95% Confidence interval**	**Fisher exact test (*P***)
**Manipulation before “Ponseti”**	Yes	10	11	0.22	(0.06–0.76)	.009
	No	38	9			
**Degree of deformation before Ponseti**	Pirani score ≤ 4	30	11	1,35	(0.41–4.43)	1.157
	Pirani score ≥ 4.5	18	9			

**Figure 3.**
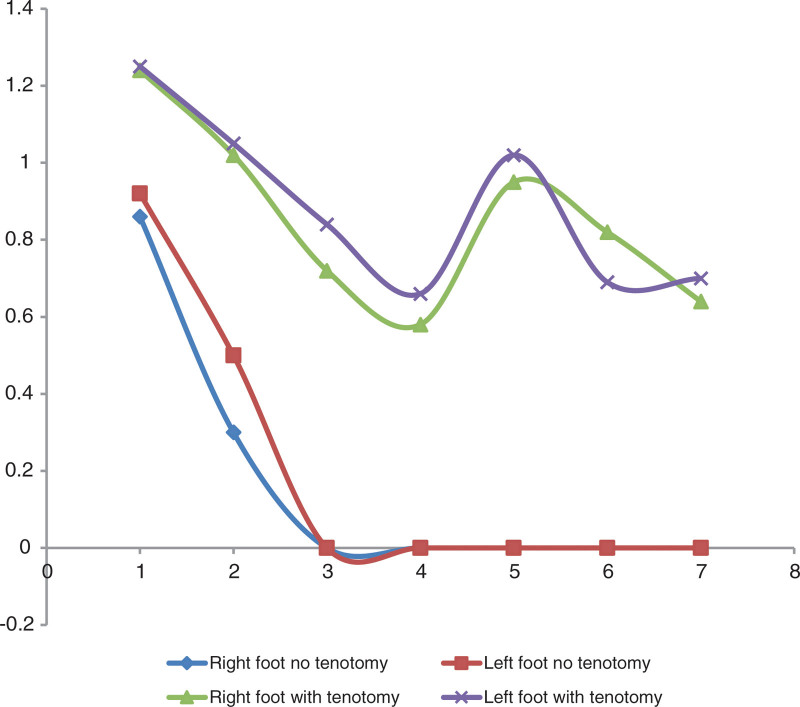
Evolutionary and comparative curves of the evolution of the Pirani score of tenotomized and nontenotomized feet We note a rapid decrease in the Pirani score of the feet which did not require a tenotomy compared to the other feet (Kolmogorov test-Smirnov: D = 0.61; *P* = .01).

**Figure 4.**
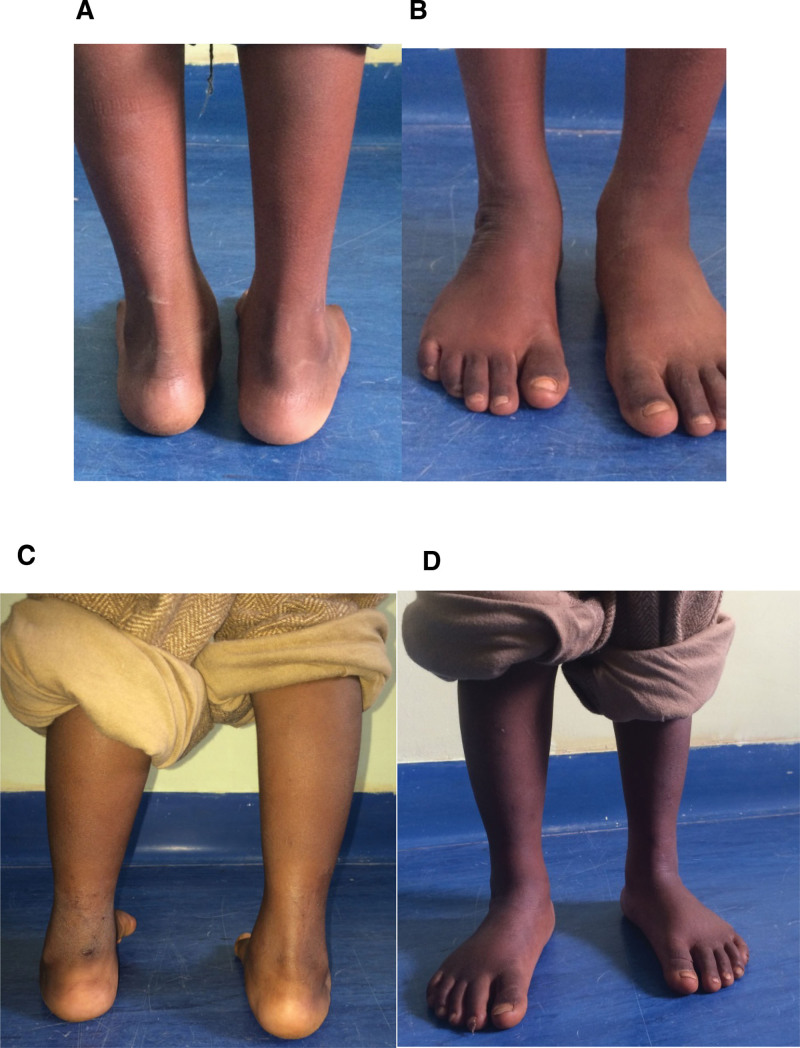
(A, B) Result after correction by Ponseti of a right unilateral clubfoot of a 5-year-old boy with the notion of anterior manipulation, which did not require a tenotomy. (C, D) Another 4-year-old boy who presented with unilateral left clubfoot without any notion of manipulation having undergone a percutaneous tenotomy.

By comparing the curve of evolution of the Pirani score of the 2 groups (tenotomized and nontenotomized) during the wearing of an abduction boot, we noted a rapidly decreasing in the Pirani score of the feet, which did not require an tenotomy compared to other feet (Kolmogorov-Smirnov test: D = 0.61; *P* = .01) (Figs. 3 and 4).

## 4. Discussion

Thanks to an increasing understanding of the relationship between the elementary deformities that constitute clubfoot, and to review of the results of different repair techniques, clubfoot management has evolved over the years.^[2]^ The first attempt to correct a clubfoot by “manipulation” was made by Hugh Owen Thomas in the 18th century.^[5]^ This correction was not codified or planned, which explained lack of favorable results. This treatment by manipulation was initialized by French orthopedists and Physiotherapists. First of all was Masse,^[6]^ and later the Parisian teams lead by Seringe in Saint-Vincent-de-Paul^[7]^ developed there technique, at also Bensahel at the Robert-Debré hospital^[8]^ and the Dimeglio team in Montpellier,^[9]^ hence the name “French method.” This method required daily handling followed by elastic band immobilization over a period of 2 months. The correction was continued for at least 6 months with 3 sessions per week. The child then wore a compression boot and a night splint until he was able to walk. It emphasized the stimulation of the evertor muscles to sustain the correction. The series by Dimeglio et al^[3]^ reported that 74% had good results. However, additional posterior release surgery was required to correct the residual deformities for a variable proportion. Indeed, the mode of physiotherapy as well as the type and nature of the rest orthosis used vary depending on the center and the operator. It is this great diversity that makes it difficult to assess the results of functional treatment. Regardless, in general the method consists of correcting in a sequential and harmonious way the deformation by first correcting the pronation and the varus before correcting the cavus of the sole of the foot and the equinism of the rear foot. The theoretical advantage of functional treatment is to allow growth while maintaining muscle tone and psychomotor development in the child.

This study concerns children of rural families who live far from the city center and the care site. Their limited financial means preclude access to conventional medical and surgical management. Consequently, they seek treatment with traditional massage therapy and only refer their children to a hospital after failure of traditional massage. Traditional massage consists of a rough manual correction of the deformity to a normal position (“manipulation”) without the use of an apparatus. This act is associated with a massage of the muscles of the lower limbs including the triceps. It is for this reason that we did not use the word “physiotherapy” but rather “manipulation” to describe traditional massage.

Our center uses the sequential plaster correction method developed by Ignacio Ponseti in 1940.^[10]^ This method consists of first correcting the cavus of the sole of the foot followed by the progressive abduction of the midfoot, which at the same time corrects the supination and the varus of the hindfoot. Equinism is reduced last, with Achilles tenotomy if necessary. The cast is changed every 5 to 7 days over a period of 4 to 8 weeks.

The advantage of this technique over functional treatment is its relative speed as well as the decreased frequency of correction sessions. The technique is well codified and easily reproducible after a period of learning. The speed of obtaining the correction must be weighed against the need for an abduction splint until the age of walking, at the cost of a recurrence proportional to the noncompliance of the wearing of the splint.^[11]^ It is for this reason that all the authors stress the need for parental understanding and buy-in on the usefulness of wearing the abduction brace.

Steinmann et al^[12]^ carried out a study on the outcome of nonsurgical clubfoot treatments. This study initially showed similar results between Ponseti and functional treatment, with 94% and 95% of patients with excellent outcomes, respectively. In terms of recurrence, 37% of the feet treated with Ponseti experienced a recurrence compared to 29% for functional treatment. The recurrences were treated surgically in the majority of cases. In the end, after a 4-year follow-up, the 2 techniques both had about 70% of patients with good outcomes and 16% with bad outcomes. The recurrences were due to the fibrous nature of the ligament tissues at the origin of the application of the foot, which makes essential the wearing of a splint that not only serves to maintain the reduction position but also acts continuously and dynamically on these tissues.^[11]^ In addition to the universally accepted medial and posterior ligament fibrosis, Kerling et al^[13]^ showed that there was also an abnormality of the extracellular matrix of the gastrocnemius muscle in congenital equinovarus clubfoot. This anomaly is the cause of muscle retraction, which plays an important role in the genesis of equinism. The connective elements of the muscles can be relaxed by regular and constant stretching exercises. The experiment carried out by Masatochi et al^[14]^ achieved this relaxation by performing daily stretching exercises of the gastrocnemius over a period of 4 weeks. After 4 weeks, they noted a lengthening of the gastrocnemius musculotendinous complex with the ability to dorsiflex more than 30°. This lengthening does not correlate with an elongation of muscle fibers, rather, it arises from relaxation of the extracellular connective tissues that surround the muscle fibers. This musculotendinous relaxation by softening the extracellular connective tissues explains the flexibility of the hindfoot of children who had benefited from regular manipulation before cast correction. The acquired flexibility makes it possible to avoid the need for surgical tenotomy.

In fact, correction by plaster alone is usually not successful in lifting the retraction of the gastrocnemius muscle. Children with a history of correction by plaster before the correction performed in the center do not present the same flexibility as those with the notion of regular handling. Tenotomy is often necessary during the Ponseti method to lengthen and lift gastrocnemius retraction.

In order to combine the advantage of the 2 techniques to improve results and reduce the need for surgery, some authors proposed the “hybrid method” they combined the Ponseti method with the French method.^[15]^ They systematically performed physiotherapy following the French method between each change of plaster. In addition, a plantar orthosis was added to the cast in order to correctly maintain the position of the sole of the foot. Their series mainly features newborns with an average age of 8 days of age. After 2 years of follow-up, they reported encouraging results with only 8% of patients requiring posterior release surgery.

Pajavani et al^[4]^ also stressed the importance of the association of Ponseti with the French method, this time with the manipulation carried out by the parents. They reported a decrease in recurrence after clubfoot correction using the Ponseti method when parents perform regular home manipulation of the splinted foot.

Another study by Smythe et al^[16]^ in Zimbabwe on the outcome of the Ponseti method showed that children with a history of previous treatment required more tenotomy compared to those who had not received previous treatment. For their series, the previous treatment always consisted of a cast correction. This result is not contradictory with ours which, for children with a history of treatment with plaster, required tenotomy at least as much as those who had no notion of previous treatment. These results lead us to reflect on the effect of cast immobilization on triceps muscle tone and the possibility of potentiation of the need for tenotomy for cast correction. The risk recurrence essentially linked to the noncompliance of the abduction splint, it is interesting to determine the effect of the combination of manipulation before or after Ponseti on this risk of recurrence.^[17]^

Unlike previous studies, our series reports the benefit of manipulation before Ponseti correction, including the effect of manipulation on gastrocnemius retraction. This concept can be used for the management of rigid clubfoot in children of walking age in order to prepare the foot for correction and reduce the need for surgery. It is obvious from previous studies, as well as ours, that the combination of functional treatment with the Ponseti method optimizes the outcome of treatment for clubfoot. This combination can occur before, during or after correction by plaster. It also involves parents by having them carry out daily and regular manipulation of the children’s feet, which can in turn be a way of getting parents involved in the care process and reduce the risk of treatment abandonment.

This study is limited by the small size of the population and its monocentric character.

## 5. Conclusion

Regular handling of a congenital equinovarus clubfoot facilitates correction by the Ponseti method. The combination of functional treatment with the Ponseti method thus makes it possible to reduce the need for surgery. The involvement of parents in this process is an attractive option to reduce abandonment and recidivism.

## Author contributions

FMR: article redaction and submission

JLA: article redaction

SK: technical help

JLG: English translation

DGS: correction
